# Future Prospect of Low-Molecular-Weight Prostate-Specific Membrane Antigen Radioisotopes Labeled as Theranostic Agents for Metastatic Castration-Resistant Prostate Cancer

**DOI:** 10.3390/molecules29246062

**Published:** 2024-12-23

**Authors:** Ratu Ralna Ismuha, Rien Ritawidya, Isti Daruwati, Muchtaridi Muchtaridi

**Affiliations:** 1 Department of Analysis and Medicinal Chemistry, Faculty of Pharmacy, Universitas Padjadjaran, Sumedang 45363, Indonesia; ratu23015@mail.unpad.ac.id; 2 Department of Pharmacy, Dharmais Cancer Hospital—National Cancer Center, Jakarta 11420, Indonesia; 3 Center for Research on Radioisotope Technology, Radiopharmaceuticals, and Biodosimetry, National Research and Innovation Agency (BRIN), South Tangerang 15314, Indonesia; rien001@brin.go.id (R.R.); isti002@brin.go.id (I.D.); 4 Research Collaboration Centre for Radiopharmaceuticals Theranostic, National Research and Innovation Agency (BRIN), Sumedang 45363, Indonesia

**Keywords:** metastatic prostate cancer, low-molecular-weight PSMA, radioisotope, imaging, therapy, theranostic

## Abstract

Prostate cancer ranks as the fourth most common cancer among men, with approximately 1.47 million new cases reported annually. The emergence of prostate-specific membrane antigen (PSMA) as a critical biomarker has revolutionized the diagnosis and treatment of prostate cancer. Recent advancements in low-molecular-weight PSMA inhibitors, with their diverse chemical structures and binding properties, have opened new avenues for research and therapeutic applications in prostate cancer management. These novel agents exhibit enhanced tumor targeting and specificity due to their small size, facilitating rapid uptake and localization at the target site while minimizing the retention in non-target tissues. The primary aim of this study is to evaluate the potential of low-molecular-weight PSMA inhibitors labeled with radioisotopes as theranostic agents for prostate cancer. This includes assessing their efficacy in targeted imaging and therapy and understanding their pharmacokinetic properties and mechanisms of action. This study is a literature review focusing on in vitro and clinical research data. The in vitro studies utilize PSMA-targeted radioligands labeled with radioisotopes to assess their binding affinity, specificity, and internalization in prostate cancer cell lines. Additionally, the clinical studies evaluate the safety, effectiveness, and biodistribution of radiolabeled PSMA ligands in patients with advanced prostate cancer. The findings indicate promising outcomes regarding the safety and efficacy of PSMA-targeted radiopharmaceuticals in clinical settings. The specific accumulation of these agents in prostate tumor lesions suggests their potential for various applications, including imaging and therapy. This research underscores the promise of radiopharmaceuticals targeting PSMA in advancing the diagnosis and treatment of prostate cancer. These agents improve diagnostic accuracy and patients’ outcomes by enhancing imaging capabilities and enabling personalized treatment strategies.

## 1. Introduction

Prostate cancer is one of the most prevalent cancers among men worldwide, ranking as the fourth most common cancer with 1,467,854 new cases reported in 2022 alone. The impact of prostate cancer on public health varies across different regions and continents. It can progress slowly within the prostate gland or spread aggressively to other parts of the body. The 2022 Globocan Data show that prostate cancer incidence and mortality rates vary significantly across continents. Europe has the highest number of new prostate cancer cases, with 473,011 cases, accounting for 32.2% of the global total. In contrast, Oceania has the lowest incidence, with 23,602 cases, representing just 1.6% of the global cases. When it comes to mortality, Asia leads with the highest number of deaths, totaling 120,485 and constituting 30.3% of global prostate cancer deaths. Oceania again has the lowest mortality rate, with 5358 deaths, which represents 1.3% of the global total [1,2]. Prostate cancer incidence rates are notably higher in transitioned countries (35.5 per 100,000) compared to those in transitioning countries (12.6 per 100,000). This disparity suggests that factors such as healthcare access, screening practices, and lifestyle may play a role in these differences. The mortality rates for prostate cancer show less variation between transitioned countries (7.3 per 100,000) and transitioning countries (6.6 per 100,000), indicating that while more cases are diagnosed in transitioned countries, the death rates are similar across different regions [1]. A study in France, published in 2020, used the French nationwide healthcare database to estimate the incidence and prevalence of metastatic castration-resistant prostate cancer (mCRPC). The study found that mCRPC accounts for 3.4% of all prostate cancer cases, with an age-standardized prevalence of 62 cases per 100,000 men and an annual incidence of 21 cases per 100,000 men. The most striking finding was the age-related trend in case prevalence, which increased from 14 cases per 100,000 in men aged 40–49 to 14,900 cases per 100,000 in men aged 90 and older. This trend underscores the significant impact of age on mCRPC. Interestingly, the corresponding incidence remained stable after the age of 70 [3]. Meanwhile, the mortality rates do not follow the same regional patterns as the incidence rates. The highest mortality rates are found in the Caribbean and Sub-Saharan Africa, which may reflect disparities in early detection and treatment [1]. Additionally, the known risk factors for prostate cancer include age, family history, genetic factors, and lifestyle factors [3].

The life expectancy of patients with metastatic castration-resistant prostate cancer (mCRPC) can vary based on factors such as overall health, the stage of cancer, the treatment response, and concurrent medical conditions. One study reported a median OS of 42.3 months for patients treated with ^177^[Lu]Lu-J591, while another analysis indicated a median OS of 17.8 months for patients receiving a different treatment ^177^[Lu]Lu-PSMA-617 every 6 weeks with intravenous 200 mg pembrolizumab every 3 weeks for a total of 2 years [4]. However, it is essential to note that this is an average estimate, and individual experiences may differ. The advances in treatment, including PSMA-targeted radioligand therapy, immunotherapy, and combination treatments, show promise in extending patients’ survival and improving their quality of life. Clinical trials and personalized treatment approaches also contribute to better outcomes for some patients [5,6]. Patients should work closely with their healthcare team to discuss their prognosis, available treatments, and supportive care in order to make an informed decision. The treatment goals for patients with mCRPC are to manage the symptoms, prevent the disease from worsening, and improve their quality of life. These advancements have provided new avenues for the treatment of mCRPC, offering patients more options and hope for improved outcomes [7].

The utilization of prostate-specific membrane antigen (PSMA) in prostate cancer cases enables highly precise imaging, targeted therapy, and personalized treatment approaches. These capabilities lead to significantly improved patient outcomes and have the potential to be applied to other types of cancer in the future [8]. PSMA is prominently and specifically expressed in prostate tissue, with the levels increasing significantly in prostate cancer cells, making it an ideal target for both the imaging and treatment of prostate cancer [9]. Due to its unique characteristics, PSMA plays a crucial role in prostate cancer diagnosis and treatment. The high specificity and abundance of PSMA on cancer cells compared to those of normal tissues allow for targeted approaches that can enhance the accuracy and effectiveness of prostate cancer management [10,11]. By leveraging the presence of PSMA, researchers and clinicians can develop innovative strategies for imaging, targeting, and treating prostate cancer with precision and efficiency [12]. PSMA plays a crucial role as a marker for hormone-independent prostate cancer and tumor neovasculature due to its distinctive expression patterns. PSMA is indeed a crucial marker for prostate cancer diagnosis and monitoring due to its high expression levels in the prostate tumor epithelium, particularly in advanced and metastatic cases [13]. Furthermore, PSMA is consistently expressed in the neovasculature of numerous solid tumors, such as adenoid cystic carcinoma (a subtype of salivary gland cancer) [5,14].

A study by Thang (2019) discovered that new treatments like [^177^Lu]LuPSMA-617 radioligand therapy may not be effective for patients with low prostate-specific membrane antigen (PSMA) expression or inconsistent FDG-avid illness. Identifying biomarkers such as PSMA expression is crucial for treatment plans for patients with metastatic castration-resistant prostate cancer (mCRPC). The study emphasized the importance of using personalized medicine techniques to enhance treatment options and patient outcomes. Further research is necessary to confirm these findings and to investigate different treatment approaches for individuals with low PSMA expression in mCRPC [15].

In addition to its role in prostate cancer, PSMA, also known as glutamate carboxypeptidase II (GCPII), is also present in various other tissues, where it helps regulate substances and their byproducts [16]. Its enzyme activity has wide-ranging effects on neuronal function and neurotransmission, particularly in breaking down N-acetylaspartylglutamate (NAAG) and affecting glutamatergic signaling. This target could potentially treat neurological disorders. Understanding the broader physiological significance of PSMA outside of its involvement in cancer biology is crucial [17,18].

In the field of theranostic, PSMA-targeting medications are crucial for the diagnosis and treatment of prostate cancer. The following are some of the ways they support the treatment of prostate cancer:

1. Diagnosis: PSMA-targeted imaging, a cutting-edge technology, can detect small or recurrent prostate cancer lesions that may not be visible on conventional imaging modalities. This breakthrough in early diagnosis and treatment planning offers a beacon of hope in the fight against prostate cancer [19,20,21,22].
2. Therapy: The revolutionary concept of theranostic combines diagnostic imaging with targeted therapy using the same PSMA-targeting agent labeled with different radioisotopes. This exact and individualized approach, tailored to each patient’s unique tumor characteristics identified through imaging, provides a reassuring level of treatment effectiveness [23,24,25].
3. Precision Medicine: PSMA-targeting agents play a pivotal role in enabling precision medicine. By identifying patients likely to benefit from targeted therapies based on the expression of PSMA on their cancer cells, this approach opens up a world of possibilities for more effective and tailored treatment strategies, inspiring a new era in prostate cancer management [20,26,27].
4. Monitoring Response: PSMA-targeted imaging can monitor treatment response over time, assess disease progression, and guide treatment modifications. Changes in PSMA expression on imaging scans can indicate the effectiveness of therapy and help in decision-making [20,28].
5. Future Directions: In the near future, cutting-edge research will revolutionize prostate cancer treatment. Ongoing studies are delving into the development of innovative PSMA-targeting agents, such as [^177^Lu]LuPSMA-617, alongside combination therapies to significantly enhance diagnosis and treatment strategies. This includes pioneering new radiotracers, examining combination therapies with immunotherapy, and dissecting resistance mechanisms. Notably, late-phase clinical trials have yielded promising results with [^68^Ga]GaPSMA-11, [^18^F]FDCFPyl, and [^99m^Tc]TcMIP-1404. Anticipated advancements in radiolabels, particularly compounds optimized for hepatobiliary excretion, will vastly improve the clinical availability of these groundbreaking treatments [29,30,31].

The wide variety of PSMA-based probes significantly contributes to the advancement of prostate cancer imaging and therapy. These probes provide clinicians with a diverse set of tools to enable accurate diagnosis, targeted treatment, and personalized care for prostate cancer patients [32]. PSMA expression in prostate cancer is a sign of a possible breakthrough because it corresponds to greater levels of serum prostate-specific antigen (PSA) and a higher Gleason score (GS) [33]. This correlation, as evidenced by studies, suggests that PSMA imaging modalities could play a significant role in the future of prostate cancer diagnosis and management. This potential impact of PSMA imaging modalities brings a sense of hope and optimism to the field, paving the way for more effective strategies [34,35]. Furthermore, PSMA could be targeted for imaging and treating the neovasculature of solid tumors due to its increased presence in the neovasculature of many solid tumors [14,36,37].

## 2. Literature Sources

This systematic review was conducted based on a literature search of PubMed and ScienceDirect databases focusing on low molecular weight and PSMA. After identifying 315 articles, 20 from PubMed and 295 from ScienceDirect, the titles and abstracts were screened, and the full texts were examined according to inclusion criteria. Following this systematic screening process, 133 relevant studies were identified. These studies were further categorized into supporting articles (29), PSMA product development (30), radiopharmaceuticals for prostate cancer (65), and preclinical data (9). These selected studies form the basis for an in-depth review of the development and use of PSMA-labeled radiopharmaceuticals in the treatment and management of prostate cancer. To ensure the quality and relevance of the studies, an exclusion process was implemented. A total of 178 studies were excluded due to being classified as “not prostate cancer or radiopharmaceutical” for lacking relevance, and an additional 4 studies were removed as redundant.

**Figure 1:**
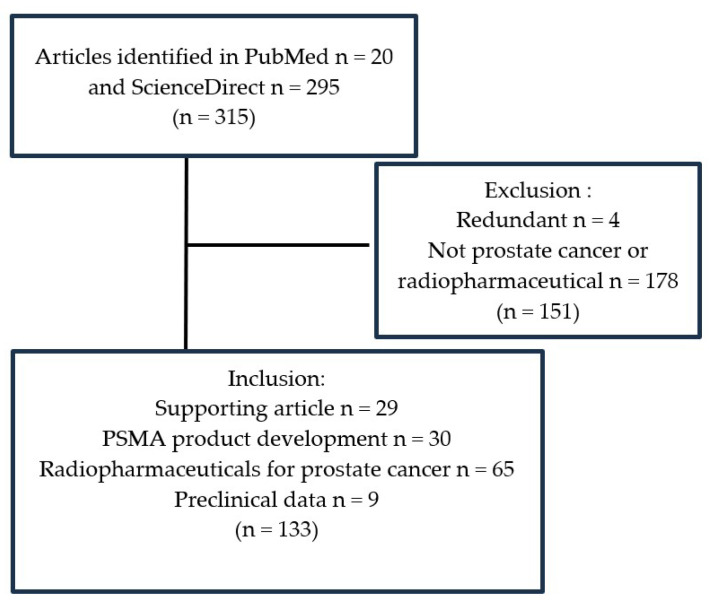
Scheme of the literature search.

As shown in Figure 1, this structured review evaluated these 133 articles to comprehensively cover the topic of PSMA-labeled radiopharmaceuticals with radioisotopes, particularly for prostate cancer. This rigorous selection process was designed to enhance the credibility of this review by focusing on studies that directly addressed the research questions concerning low-molecular-weight PSMA labeled with radioisotopes.

## 3. Low-Molecular-Weight PSMA

**Figure 2:**
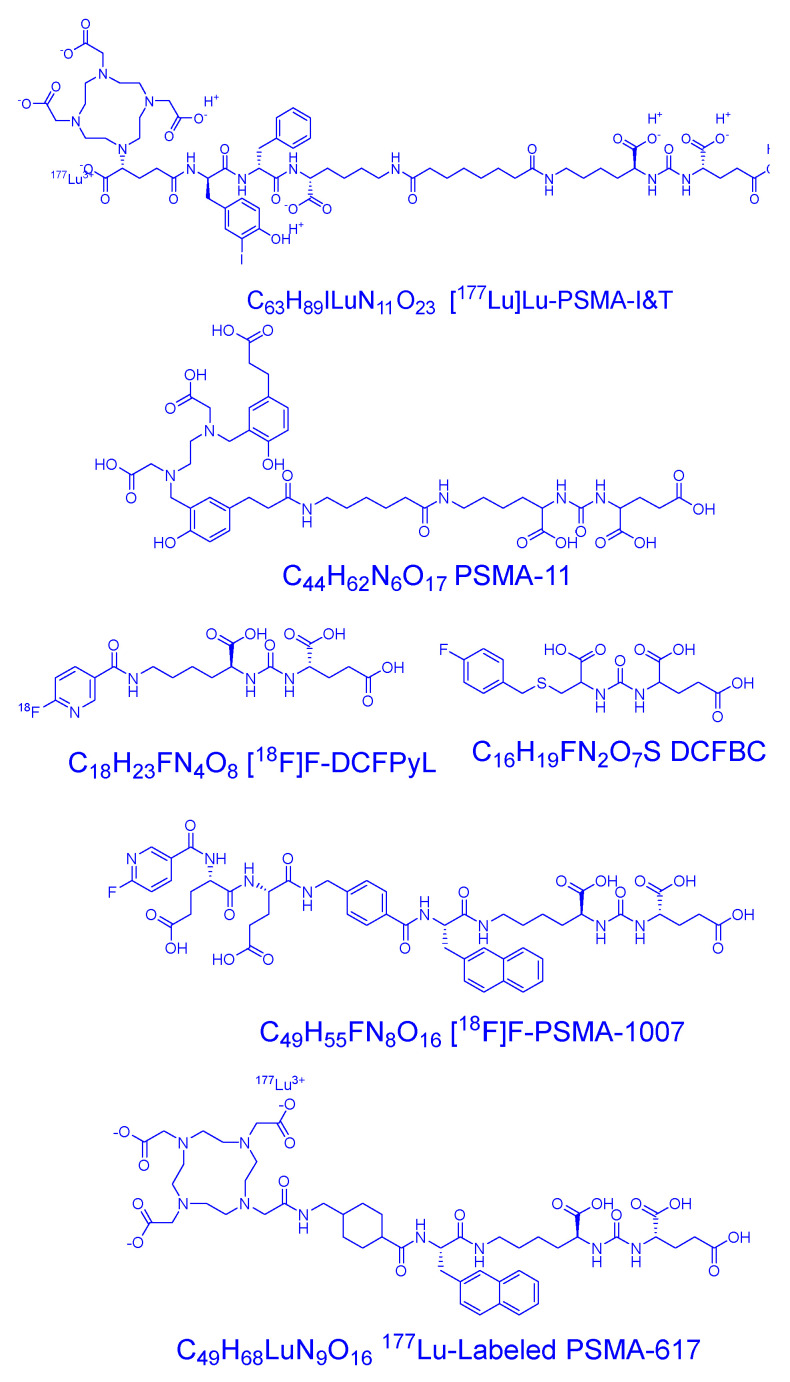
Comparison of different PSMA ligands.

Low-molecular-weight PSMA inhibitors have been the subject of considerable attention in nuclear medicine owing to their distinct advantages over monoclonal antibodies. These inhibitors, including [^18^F]F-DCFPyL, [^18^F]F-PSMA-1007, [^68^Ga]Ga-PSMA-HBED, [^177^Lu]Lu-PSMA-617, [^177^Lu]Lu-PSMA-I&T, [^99m^Tc]Tc-MIP-1404, and [^99mTc^]Tc-PSMAI&S, demonstrate high affinity to PSMA and rapid tumor uptake with quick excretion via the kidneys [38]. PSMA, also known as glutamate carboxypeptidase II (GCPII), is a promising target for prostate cancer imaging and therapy due to its overexpression in advanced and metastatic prostate cancer cells. Associated inhibitors are illustrated in Figure 2 [39,40].

The development of PSMA-targeted radiopharmaceuticals, including small-molecule anti-cancer drug conjugates, peptides, modified antibodies, radiotherapy, and immunotherapy, holds promise for novel therapeutic approaches in prostate cancer [37,41,42,43]. In the practical field of nuclear medicine, PSMA-targeted radiopharmaceuticals have been applied in imaging and therapy for prostate cancer patients. Their potential in treating oligometastatic prostate cancer is a significant breakthrough. Furthermore, comparing different imaging modalities, such as [^99m^Tc]Tc-PSMA scintigraphy and [^68^Ga]Ga-PSMA PET/CT, has been studied to detect metastatic prostate cancer, emphasizing the importance of selecting the most appropriate imaging technique [44].

The unique chemical structures and binding characteristics of low-molecular-weight PSMA inhibitors show promise for advancing our understanding and treatment of prostate cancer. They fall into several categories, each with particular uses and advantages:

1. Urea-Based Compounds: Among the first designed to inhibit PSMA activity, these inhibitors demonstrate high precision and specificity. Their urea moiety interacts with the zinc active site of PSMA, a key component for its hydrolytic activity, effectively inhibiting PSMA. This high affinity and specificity for PSMA reassures their potential in developing radiopharmaceuticals for prostate cancer imaging [7].
2. Phosphonates, Phosphates, and Phosphoramidates: These are other classes of low-molecular-weight PSMA inhibitors that interact with the zinc active site of PSMA. They contain phosphorus-based functional groups, contributing to their binding affinity and specificity for PSMA [38].
3. Glu-Ureido-Based Peptides: Peptides bearing the Glu-Urea-Lys and Glu-GABA-Asp pharmacophore have emerged as a promising avenue for research due to their ability to target PSMA receptors in prostate cancer. These peptides have shown high binding affinity to PSMA-positive cells and specific accumulation in prostate tumor lesions, making them promising candidates for molecular imaging and therapy [45].

Glu-Ureido-based peptides, with their Glu-Urea-Lys pharmacophore, have been extensively studied for their potential in targeting PSMA receptors in prostate cancer [46]. These peptides have demonstrated encouraging outcomes, especially in the early diagnosis of prostate cancer, to find significant potential to enhance patient outcomes. The goal of designing and synthesizing peptides based on Glu-Ureido was to produce peptides that would specifically accumulate in prostate tumor lesions, have a high binding affinity to PSMA-positive cells, and be stable in biological conditions [47]. Research has shown that radiolabeled Glu-Ureido-based peptides for prostate cancer imaging and treatment can effectively target PSMA receptors. Peptides containing Glu-Urea-Lys and Glu-GABA-Asp pharmacophores have shown efficient interaction with the crystal structure of PSMA, high binding energies, and strong binding affinity to PSMA-positive cells [48]. These peptides have been radiolabeled with ^99m^TC and evaluated for their in vitro and in vivo characteristics, including stability, affinity towards PSMA-positive cells, biodistribution, and imaging capabilities. Consequently, Glu-Ureido-based peptides, such as EUK-G-A-D-NA-[^99m^Tc]Tc-HYNIC, were observed to have high affinity to PSMA, good stability, and specific accumulation in prostate tumor lesions. These characteristics make them highly promising candidates for targeted imaging and therapy in prostate cancer, representing a significant advancement in the field of molecular imaging and targeted therapy for this disease [45]. The maximum molecular weight of PSMA considered in the context of targeting therapies is typically around 1–2 kDa for low-molecular-weight compounds, such as Glu-ureas targeting the catalytic domain of PSMA. This range has revived interest in developing small-molecule PSMA-targeting agents [49].

**Figure 3:**
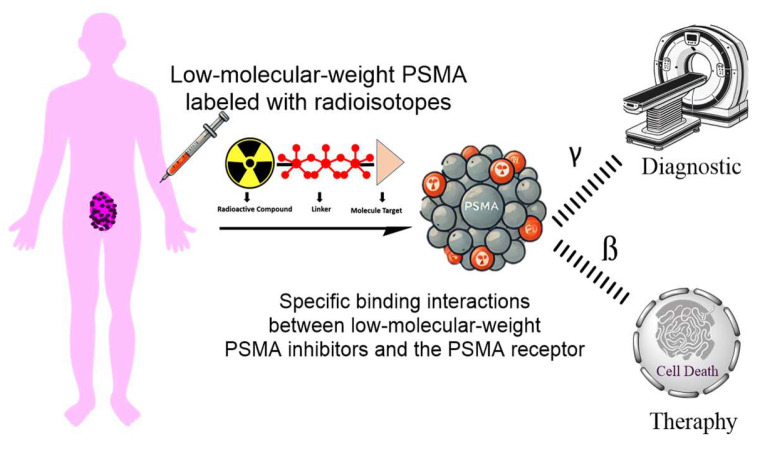
The novelty of radioisotope-labeled low-molecular-weight PSMA inhibitors as mCRPC theranostic drugs.

The specific binding interactions between low-molecular-weight PSMA inhibitors and the PSMA receptor are essential for their diagnostic and therapeutic applications in prostate cancer [50]. Targeting cells and tissues that express PSMA depends on these interactions, as illustrated in Figure 3. The details about how low-molecular-weight PSMA inhibitors interact with the receptors are mentioned below:

1. The active site of PSMA is typically targeted by low-molecular-weight inhibitors like phosphonates (PSMA-1007), urea-based compounds (PSMA-617), and peptides (Glu-GABA-Asp). The active site’s zinc ions interact with functional groups in these inhibitors, which is essential for the enzyme’s catalytic activity [51].
2. Low-molecular-weight PSMA inhibitors are meticulously designed, showcasing incredible advances in science. These precisely crafted inhibitors bind to overexpressed PSMA receptors on the surface of prostate cancer cells, enabling targeted imaging and therapy [52].
3. Pharmacophore Interactions: The peptides in low-molecular-weight PSMA inhibitors, consisting of Glu-Ureido-Lys or Glu-GABA-Asp pharmacophores, engage in specific binding sites on the PSMA receptor. These interactions, which are the foundation of their high binding affinity and selectivity, provide a deep understanding of the scientific basis of these peptides’ effectiveness towards PSMA-positive cells [52].
4. Electrostatic and Hydrophobic Interactions: Low-molecular-weight PSMA inhibitors engage in electrostatic and hydrophobic interactions with the PSMA receptor, contributing to their binding affinity and stability. These interactions play a role in the inhibitors’ recognition and binding to the receptor [10].
5. The ingenious design of some low-molecular-weight PSMA inhibitors triggers receptor-mediated endocytosis, a mechanism that leads to the internalization of the inhibitor along with the PSMA ligand into the cell. This internalization mechanism holds immense potential in significantly enhancing the therapeutic efficacy of the inhibitors by delivering the drug payload directly to the target cells, offering a beacon of hope in the fight against prostate cancer [53].

As shown in Figure 3, low-molecular-weight PSMA inhibitors represent a promising class of compounds in the fight against prostate cancer. Their ability to selectively target the PSMA receptor, trigger receptor-mediated endocytosis, and facilitate the direct delivery of therapeutic agents positions them as valuable tools in both treatment and diagnostic strategies for prostate cancer management. Understanding the receptor interactions of low-molecular-weight PSMA inhibitors is crucial for advancing our efforts against prostate cancer. These interactions are also essential for developing targeted imaging agents and therapeutic strategies [10].

## 4. Radionuclide Characteristics

Understanding the characteristics of radionuclides is essential for their safe and effective use in different applications, particularly in medicine and research, where precise control of radiation exposure and energy is critical [54]. The radionuclides used in cancer therapy possess specific characteristics that make them suitable for therapeutic applications. Some key characteristics include the following:

1. Physical Half-Life.
  The ideal half-life for therapeutic radionuclides typically ranges from hours to days to ensure effective treatment. A radionuclide’s physical half-life determines the length of its radioactive decay [55].
2. Radiation Energy.
  The energy and type of radiation that a radionuclide releases (e.g., alpha, beta, gamma) impact how well it penetrates tissues and how effectively it treats cancer cells [56].
3. Production Method.
  The method of radionuclide production, whether through cyclotrons, reactors, or generators, impacts the availability and practicality of using radionuclides in clinical settings [55].
4. Daughter Products.
  Some radionuclides decay into daughter products that may have different chemical or physical properties, affecting the overall behavior and safety of the radiopharmaceutical [52].
5. Radionuclide Purity.
  The purity of the radionuclide, including the absence of impurities or contaminants, is essential to ensure the radiopharmaceutical’s safety and efficacy in cancer therapy [55].
6. Targeting Specificity.
  The ability of a radionuclide to specifically target cancer cells or tumor biomarkers, such as PSMA in prostate cancer, is crucial for maximizing therapeutic effects while minimizing off-target effects [10,57].
7. Therapeutic Range.
  The therapeutic range or the range of radiation emitted within tissues determines the radionuclide’s ability to deliver a therapeutic dose to the target tumor while sparing surrounding healthy tissues [56].

It is crucial to understand the characteristics of radionuclides when designing effective radiopharmaceuticals for cancer therapy, optimizing treatment outcomes, and ensuring patient safety [58]. Furthermore, these emitters play crucial roles in various applications, from cancer therapy to diagnostic imaging. Alpha emitters are known for their high potency and short range, beta emitters are effective against medium to large tumors, and gamma emitters are widely used in imaging techniques. Understanding their characteristics is essential for their targeted and practical application in medical settings, as summarized in Table 1 [43,59].

The synthesis of ^68^Ga-labeled PSMA compounds often involves a chelation process where ^68^Ga is complexed with a bifunctional chelator (BFC) that is attached to the PSMA-targeting ligand. Common BFCs include HBED-CC or DOTA. The process typically involves the preparation of the ligand while the PSMA-targeting ligand is synthesized and functionalized to include a chelator. After that, the process continues with radiolabeling ^68^Ga, eluted from a generator, and mixed with the ligand under specific conditions (e.g., pH, temperature) to facilitate the formation of the radiolabeled compound. The final product is purified to remove unreacted ^68^Ga and other impurities using the reliable methods of HPLC or solid-phase extraction [17]. ^18^F-labeling typically involves a nucleophilic substitution reaction where a precursor containing a leaving group is reacted with ^18^F. The PSMA ligand is synthesized with a suitable leaving group (e.g., tosylate or mesylate). After that, the precursor is reacted with ^18^F in a suitable solvent at elevated temperatures to facilitate the substitution reaction. The purification often uses HPLC to ensure the quality and reliability of the final product [77].

Several methods are essential to identify radiolabeled drugs that target prostate-specific membrane antigen (PSMA) to evaluate their specificity and effectiveness. Immunohistochemistry (IHC) uses radiolabeled antibodies to identify PSMA expression in tissue samples and establish the target’s presence in malignancies. Another crucial method for assessing the stability and purity of radiolabeled substances is high-performance liquid chromatography (HPLC) and radio-thin-layer chromatography (RTLC), which separates them from unreacted precursors and byproducts to guarantee the given dose. In order to successfully label PSMA-targeting medicines, mass spectrometry (MS) is also essential for describing the molecular weight and structure of radiolabeled molecules [78]. Biodistribution studies are conducted to better understand the pharmacokinetics and targeting efficacy of radiolabeled PSMA medicines. These studies involve administering radiolabeled PSMA compounds to animal models and measuring the distribution of radioactivity in various tissues over time [79].

**Figure 4:**
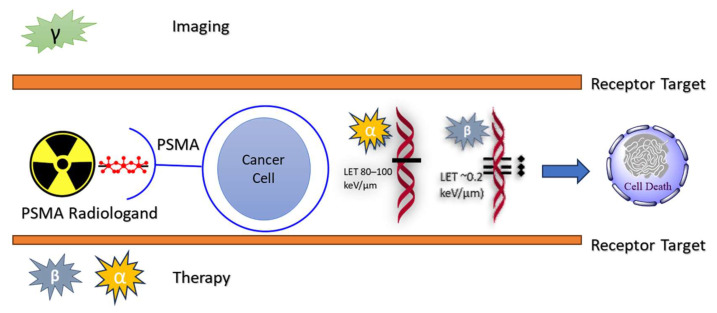
Mechanism of radiation in cancer lesions.

As shown in Figure 4, the targeted delivery of therapeutic isotopes straight to prostate cancer cells is the method of radiation in cancer lesions using gamma and beta emitters labeled for prostate-specific membrane antigen (PSMA). PSMA is a molecular target for radiolabeled ligands that bind selectively to malignant cells, which tend to overexpress it, in prostate cancer [50]. These ligands help with positron emission tomography (PET) imaging by conjugating with gamma emitters like Gallium-68, which enables accurate tumor localization [80]. On the other hand, beta emitters, such as ^177^Lu, provide therapeutic radiation that, when internalized by the cancer cells, causes cytotoxicity. The targeted therapy uses beta particles to enter the tumor tissue, break DNA, and cause cell death while avoiding harm to nearby healthy tissues. PSMA-targeted radioligand therapy is a promising treatment option for prostate cancer because of its dual approach, which increases both therapeutic efficacy and diagnostic accuracy. 

Both alpha and beta particles are used in cancer therapy because the radiation emitted is able to kill cancer cells [4,78]. Alpha particles have a high linear energy transfer (LET) of approximately 80–100 keV/μm, which means they deposit a large amount of energy over a very short range (40–100 μm). This makes them particularly effective for targeting small tumors or single cancer cells, as they can cause significant damage to the DNA of cancer cells while sparing surrounding healthy tissue. In contrast, beta particles have a lower LET (~0.2 keV/μm) and a longer mean tissue range (1–5 mm). This allows beta emitters to irradiate larger tumor volumes, making them suitable for treating larger or heterogeneous tumors. The cross-fire effect of beta particles can provide a more uniform dose distribution within the tumor [81].

## 5. PSMA Labeled with Radioisotopes

Research on low-molecular-weight PSMA (prostate-specific membrane antigen) inhibitors labeled with radioisotopes for the treatment of prostate cancer has generated significant interest in the fields of nuclear medicine and oncology. These compounds, known as radioligands, are crucial for both diagnosing and treating prostate cancer. Although there have been initial difficulties with target selectivity and reproducibility during synthesis, low-molecular-weight (LMW) conjugates allow flexibility in structural change and a decreased danger of immune responses. For medications with short half-lives, their shorter circulation durations may be helpful, but renal clearance and possible buildup in particular organs should be taken into account [77]. Next, we describe some critical points based on research findings in this area.

### 5.1. Imaging Applications

Imaging techniques play a crucial role in managing prostate cancer patients undergoing PSMA-targeted radioligand therapy by aiding in diagnosis, treatment planning, and monitoring treatment response [82,83]. These techniques are used to visualize and assess prostate cancer lesions with precision and accuracy through imaging techniques such as positron emission tomography (PET) or single-photon emission computed tomography (SPECT). This allows for precise localization of tumors and assessment of treatment responses [84]. In addition, noninvasive molecular imaging can assess dynamic changes in the tumor microenvironment over time, providing real-time information about biological processes and treatment responses, which is often not possible with conventional imaging techniques [85]. The imaging application findings are listed in Table 2.

**Figure 5:**
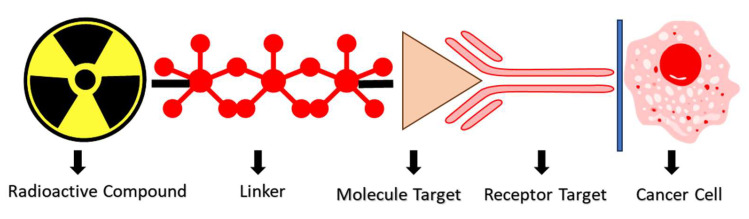
Schematic representation of a radioactive compound targeting cancer cells.

Figure 5 shows how a radioactive compound is strategically used in imaging or therapy to target cancer cells precisely [95]. It is expertly attached to a targeting molecule, such as an antibody or small molecule, through a chemical bridge known as a linker [96]. This targeting molecule specifically and effectively binds to a receptor on the cancer cell’s surface. The receptor confidently guides the radioactive compound to its intended location, where it can assert its therapeutic or imaging effect on the cancer cell. This precise interaction allows for the optimal and confident diagnosis or treatment of the disease [4,78].

### 5.2. Therapeutic Applications

There is a growing interest in combining PSMA-targeted radioligand therapy with other treatment modalities, such as immunotherapy and androgen receptor-targeted therapies, as detailed in Table 3. This combination approach may enhance treatment outcomes and overcome resistance mechanisms in mCRPC [44]. Radiolabeled PSMA ligands are utilized in therapeutic applications to specifically treat prostate cancer. These therapeutic applications underscore the precision and effectiveness of PSMA-targeted radioligand therapy in treating prostate cancer patients, offering a promising outlook for the condition’s treatment [97,98].

In the past three years, prostate-specific membrane antigen (PSMA) has become a well-established target for imaging and therapy in prostate cancer, and the isotopes ^43^Sc and ^47^Sc have been explored for their potential in PSMA-targeted radioligand therapy [52,108]. ^43^Sc is particularly noted for its favorable properties in clinical applications, including a half-life of 3.89 h and positron emission, which is suitable for imaging purposes. It has demonstrated PSMA-specific uptake and high complex stability when used with PSMA-targeting ligands. A crucial feature that informs the audience about the possible advantages of this isotope and its promising role in pretherapy planning is that it demonstrates dosimetry advantages over conventional isotopes like ^177^Lu. Its promise in clinical settings is further supported by its distribution kinetics comparable to those of ^177^Lu-PSMA-617 [108].

On the other hand, ^47^Sc is being investigated as a new radiometal β-emitter for PSMA-targeted therapy. With a half-life of 3.35 days, ^47^Sc allows for extended treatment regimens and is considered beneficial for enhancing the effectiveness of radioligand therapies, particularly in the radiotheragnostic paradigm, when combined with other isotopes to improve therapeutic efficacy [52,66]. The importance of continuing research in this ever-evolving subject is that it is essential to developing new radioligands and isotopes that will improve treatment options for patients with metastatic prostate cancer. Both isotopes are part of this exciting journey, contributing to developing bifunctional chelators that can rapidly and stably complex these metal ions, which are crucial for minimizing off-target toxicity and maximizing therapeutic effects in prostate cancer treatment [52,108,109].

### 5.3. In Vitro Studies Involving Cell Lines and PSMA

In vitro studies play a crucial role in biomedical research, providing valuable insights into basic biological processes, disease mechanisms, drug development, and personalized medicine approaches. These studies are often a critical step in the research pipeline before advancing to more complex in vivo studies or clinical trials [70,110]. Various prostate cancer cell lines, including LNCaP, PC3, and DU145, are commonly used in in vitro studies to investigate PSMA expression and function. These cell lines provide models to study the biology of prostate cancer and the role of PSMA in tumor progression and metastasis [110].

LNCaP is a human prostate cancer cell line that is commonly used in research to study prostate cancer biology and treatment. It is particularly notable for expressing prostate-specific membrane antigen (PSMA), making it a valuable model for evaluating PSMA-targeted therapies and imaging agents. LNCaP cells, specifically the C4-2 subline, were utilized for competitive cell binding assays and internalization studies to assess the efficacy of the developed labeled PSMA ligands [111]. LNCaP cells are unique in their ability to model various stages of prostate cancer progression, from androgen dependence to castration resistance and even the transition to neuroendocrine prostate cancer (NEPC) features. This makes them invaluable for studying the disease’s evolution and treatment responses [110]. In addition to in vitro studies, LNCaP cells are often inoculated into immunocompromised mice to create xenograft models. This allows researchers to assess tumor growth, treatment responses, and the biodistribution of radiopharmaceuticals in a living organism [92]. The use of LNCaP cells in both in vitro and in vivo settings helps in understanding the pharmacokinetics and biodistribution of radiopharmaceuticals, which is essential for optimizing therapeutic strategies and improving patient outcomes [17,109].

DU145 cells are derived from a human prostate carcinoma and are known for their androgen independence, making them a valuable model for studying advanced prostate cancer that does not respond to hormone therapy. This characteristic allows researchers to investigate therapeutic strategies for more aggressive forms of the disease [66,112]. DU145 cells are frequently used in studies involving various therapeutic agents, including radiopharmaceuticals. They provide a platform for evaluating the efficacy of targeted therapies, such as those utilizing PSMA or other prostate cancer-specific antigens [66].

The PC3 cell line is derived from a human prostate adenocarcinoma and is known for being androgen-independent. This characteristic makes PC3 cells particularly useful for studying advanced prostate cancer, especially in cases where the cancer has progressed beyond hormone therapy. PC3 cells are utilized in various studies, including those focused on the development and evaluation of targeted therapies, such as radiopharmaceuticals. They are often employed to assess the efficacy of treatments that target specific receptors or antigens associated with prostate cancer. By conducting in vitro studies, researchers can gain valuable insights into the mechanisms of action of potential cancer treatments, evaluate their specificity and efficacy, and guide the development of novel therapeutic strategies for cancer patients in a controlled environment [113]. The transition from in vitro studies to in vivo applications often reveals discrepancies in ligand performance. Validating the affinity and efficacy of ligands in animal models and eventually in human trials is essential but can be fraught with challenges related to biological variability and tumor microenvironment interactions [114].

### 5.4. Clinical Studies

The use of radionuclides as theranostic agents allows for the simultaneous diagnosis and treatment of cancer. This approach enables targeted therapy that can directly affect tumor cells while minimizing damage to healthy tissues [115]. Research and trials are being conducted to evaluate the safety, effectiveness, and potential applications of radiolabeled PSMA ligands in diagnosing and treating prostate cancer.

As shown in Table 4, the effectiveness and widespread use of PSMA-targeted radiopharmaceuticals in clinical practice for diagnosing and treating prostate cancer are clearly associated with these characteristics. Future research is likely to focus on optimizing the pharmacokinetics and biodistribution of low-molecular-weight PSMA ligands, improving their targeting efficiency, and minimizing off-target effects [116].

### 5.5. Linkage Used in PSMA Synthesis Radiolabeled

The process of producing radiolabeled prostate-specific membrane antigen (PSMA) ligands for imaging or therapy involves strategically chosen linkers to accurately attach a radioactive label to the targeted molecule [43]. The stability, pharmacokinetics, and targeting efficacy of the radiolabeled PSMA ligands critically depend on the careful selection of these linkers. Some of the common linkers used in PSMA synthesis for radiolabeling are listed in Table 5.

The linkers play an essential role in effectively synthesizing and radiolabeling PSMA ligands for a variety of nuclear imaging and therapy applications. The selection of a linker depends on factors such as the radioisotope used, desired stability, pharmacokinetics, and specific requirements of the radiolabeled PSMA ligand. The pharmacokinetics of these ligands are tailored to ensure rapid accumulation in tumors and prolonged retention for therapeutic effects. This is crucial because the ideal characteristics for imaging (quick clearance from non-target tissues) differ from those needed for therapy (high tumor uptake and retention) [108].

## 6. Potential Side Effects of Low-Molecular-Weight PSMA Ligands Labeled with a Radioisotope

The use of low-molecular-weight PSMA radioligands in prostate cancer treatment has shown promising results. However, there could be potential side effects and safety considerations that need to be addressed [123]. Some of the critical points regarding safety considerations and potential side effects of low-molecular-weight PSMA ligands labeled with a radioisotope are detailed in Table 6.

It is crucial for healthcare providers to carefully assess the risk–benefit profile of using low-molecular-weight PSMA radioligands in prostate cancer treatment and to closely monitor patients for any potential side effects or adverse reactions. Individualized treatment plans and multidisciplinary care are essential to optimize patient outcomes and safety [9]. Furthermore, radiopharmaceuticals generally have a relative lack of toxicity compared to traditional cytotoxic and cytostatic drugs, making them a safer option for elderly patients who may have diminished physiological reserves and increased sensitivity to adverse effects [125]. By focusing on targeted therapies, personalized medicine aims to reduce side effects and improve the overall safety of treatments. This is particularly important for older patients or those with comorbidities who may not tolerate conventional therapies well [126].

## 7. Future and Prospective Views of Authors

Enhancing the affinity for PSMA in ligand design is crucial to developing effective targeted prostate cancer therapies. Researchers face several challenges in this process, which they aim to address to improve the efficacy of PSMA-targeted therapies. PSMA-based probes offer several advantages for prostate cancer imaging and therapy, but they also come with certain limitations. The advantages and drawbacks clearly demonstrate the challenges involved in developing and applying PSMA-based probes for prostate cancer detection and treatment, emphasizing the imperative for sustained investigation and development [105].

The specific challenges in enhancing the affinity for PSMA in ligand design are summarized along with references to previous studies (Table 7). Addressing these challenges in ligand design through innovative strategies, structural optimization, and a deep understanding of PSMA biology is essential to enhance the affinity, specificity, and therapeutic potential of PSMA-targeted therapies for prostate cancer and other PSMA-expressing malignancies. Through creative thinking and structural optimization, we can greatly enhance the effectiveness, specificity, and therapeutic potential of PSMA-targeted drugs [116]. However, it is crucial to acknowledge the broader challenges and complexities associated with cancer treatment. Addressing critics’ concerns may involve a multifaceted approach that integrates advancements in molecular targeting with a deeper understanding of cancer biology, personalized medicine strategies, and the development of combination therapies to maximize the impact of PSMA-targeted treatments in clinical practice [44,77]. These directions reflect a broader trend towards precision oncology, where treatments are increasingly tailored to the individual characteristics of patients and their tumors [127].

The process of introducing a radiolabeled imaging agent into clinical use is a complex and meticulous endeavor. It commences with the identification and development of the [127] imaging agent, involving intricate chemistry and precise radiolabeling work. After successful chemistry and radiolabeling, preclinical testing is conducted to evaluate the agent’s efficacy and safety. Investigators, with their deep understanding of the clinical trial design, play a crucial role in validating the molecular imaging agent, including safety and efficacy endpoints. Pilot studies are then conducted to activate the Investigational New Drug (IND) application. The imaging agent must undergo regulatory review and approval, a process that demands rigorous scrutiny. Once approved, the imaging agent can be commercialized and made available to healthcare providers and patients. This complex process underscores the importance of collaboration among researchers, clinicians, regulatory agencies, and industry partners, all working together to successfully translate a radiolabeled imaging agent from discovery to clinical use [134].

It is imperative to comprehensively analyze current research limitations to enhance the discussion significantly. The study of prostate-specific membrane antigen (PSMA) and its ligands is vital for identifying potential biases and resolving conflicting results in the literature. Bias can manifest in common areas, and contradictory findings can arise from various sources:

1. Selection Bias: Studies may have biases in patient selection, particularly if they focus on specific populations (e.g., only patients with advanced prostate cancer). This can limit the generalizability of the findings to broader patient populations. For instance, if a study only includes patients with high PSMA expression, the results may not apply to those with lower expression levels [80].
2. Biological Variability: Because prostate cancer is a diverse illness, PSMA expression can fluctuate significantly between people and even within a single patient’s tumor. This biological diversity may give rise to inconsistent outcomes regardless of the effectiveness of PSMA-targeting drugs. Tumor grade, stage, and other biomarker presence are examples of factors that can affect PSMA expression and, in turn, the results of imaging and therapy [105].
3. Publication Bias: Positive results are more likely to be published than negative or inconclusive findings. This can lead to an over-representation of successful ligand designs or therapeutic outcomes in the literature, skewing the perceived efficacy of PSMA-targeted therapies [67].
4. Methodological Variability: Differences in study design, including variations in ligand synthesis, labeling techniques, and imaging modalities, can lead to conflicting results. For example, studies using different radionuclides or imaging techniques may report varying levels of tumor uptake and retention, complicating comparisons [108,116].
5. Confounding Variables: Concurrent therapies, tumor heterogeneity, and patient demographics are a few more variables that may affect study results. Inadequate control over these confounding factors may result in inaccurate results regarding the efficacy of ligands that target PSMA [108].
6. Influence of the Industry: Research sponsored or conducted by pharmaceutical companies may be biased, mainly if intended to promote a specific product. Conflicts of interest may arise, potentially affecting a study’s design, data interpretation, and result reporting. For this reason, the Declaration of Interests should be addressed after the publication [79].
7. Technological Developments: Older research may become obsolete due to the quick development of imaging and treatment technology. Compared to earlier research, new methods may uncover unknown facets of PSMA biology or ligand interactions, producing contradicting results [53].

Future studies focused on optimizing therapeutic medicines for metastatic castration-resistant prostate cancer (mCRPC) should focus on a few critical areas to increase therapy efficacy and safety. This research should focus on creating new ligands or altering existing ones to improve tumor targeting and decrease off-target effects, which may help reduce toxicity [131]. Furthermore, interactions between PSMA-targeted radionuclide therapy and other therapeutic modalities, such as immune checkpoint inhibitors or novel hormonal agents, could provide information about synergistic effects that can improve overall treatment outcomes. These combinations will then be assessed by clinical trials for efficacy and safety, benefiting patients [132]. Therefore, understanding pharmacokinetics and biodistribution for new agents is essential because it will show how an agent’s chemical structure changes its distribution throughout the body, tumor retention, and removal from healthy tissues [131]. The clinical translation of PSMA-targeted radiopharmaceuticals faces several challenges, including cost, accessibility, and long-term effects on patients. The development and production of PSMA-targeted radiopharmaceuticals can be expensive. It includes the costs of synthesizing radioligands, quality control, and regulatory compliance. In addition, specialized facilities and equipment for radiopharmaceutical production and administration are also essential to prioritize [26]. Furthermore, healthcare providers require continuous education and training on using PSMA-targeted radiopharmaceuticals, including aspects of their administration, potential side effects, and how to manage complications. It is crucial to ensure their safe and effective use in clinical practice [20].

In conclusion, while the literature on PSMA-targeted therapies is rich and informative, critically evaluating the potential biases and conflicting findings is essential. A comprehensive understanding of these factors can help guide future research and clinical applications in prostate cancer treatment. Many obstacles to developing low-molecular-weight PSMA-labeled radioisotopes require careful thought and calculated preparation [49,52]. Addressing regulatory barriers, streamlining production procedures, and recognizing patient variability are critical to progress in this field. To overcome these obstacles and enhance the practical application of PSMA-targeted medicines, a comprehensive strategy involving cooperation between researchers, physicians, and regulatory agencies would be necessary. Further research and innovation could lead to improvements in the diagnosis and treatment of prostate cancer [20,133].

## 8. Conclusions

Radiopharmaceuticals targeting PSMA have displayed significant promise in advancing prostate cancer diagnosis and treatment. They enhance imaging capabilities, enable personalized treatment strategies, and improve diagnostic accuracy. Healthcare providers aim to achieve better outcomes for prostate cancer patients by embracing innovative therapies through rigorous clinical trials and research. This entails raising life expectancy, improving quality of life, and paving the way for the complete or long-term eradication of the illness. A more defined course for future research can be set by identifying these particular research areas and suggesting joint efforts among researchers. Such focused research projects will benefit PSMA-targeted therapy development and the field’s general understanding of prostate cancer treatment. Ultimately, this will improve patient outcomes and more effective medical procedures. 

## Figures and Tables

**Table 1:** The characteristics of radionuclides utilized in nuclear medicine services.

Radionuclide	Half-Life	Emission Type	Maximum Energy	Application	Reference
^68^Ga	67.7 min	β^+^	1900 keV	PET imaging of tumors, including prostate cancer	[17,60,61,62].
^99m^Tc	6.0 h	γ	140 keV	SPECT imaging for various medical conditions	[63,64].
^111^In	2.8 days	EC	245 keV	Targeted radionuclide and imaging applications	[61,65].
^177^Lu	6.7 days	β^−^/γ	497 keV	Therapeutic radiopharmaceutical for cancer	[49,66].
^211^At	7.2 h	α	6790 keV	Targeted alpha particle therapy	[67,68].
^225^Ac	10.0 days	α/β^−^	6830 keV	Alpha particle therapy for cancer	[61,66,69].
^213^Bi	45.6 min	α/β^−^	8320 keV	Alpha particle therapy for targeted treatment	[66,70].
^67^Cu	2.6 days	β^−^/γ	575 keV	Therapeutic applications in cancer treatment	[71,72].
^131^I	8.0 days	β^−^, γ	606 keV	Thyroid cancer therapy and imaging	[43,73].
^125^I	59.4 days	γ	35.5 keV	Brachytherapy for malignant tumors	[43,73].
^124^I	4.18 days	β^+^, γ	1500 KeV (β^+^,) and 602 keV (γ)	Used in PET imaging, particularly for radioimmunotherapy and imaging of prostate cancer	[74].
^18^F	109.8 min	β^+^	634 KeV	PET imaging for various medical conditions	[63,75].
^45^TI	3.08 h	β^+^	439 KeV	PET imaging, particularly for targeting prostate-specific membrane antigen (PSMA) in prostate cancer	[76].
^90^Y	2.7 days	β^−^	2284 keV	Therapeutic radiopharmaceutical for cancer	[66].
^47^Sc	3.4 days	β^−^/γ	162 keV	Therapeutic radiopharmaceutical for cancer	[66].

**Table 2:** Imaging applications.

Radiopharmaceutical	Findings	Reference
[^68^Ga]Ga-PSMA PET/CT	High specificity and sensitivity in identifying lesions related to primary and metastatic prostate cancer. More sensitive than conventional imaging techniques. High diagnostic accuracy in identifying prostate cancer that has returned and metastasized to lymph nodes.	[38,80].
[^68^Ga]Ga-IPSMA-BN	Promising results in healthy volunteers with no side effects. Rapid clearance from the bloodstream. Low internal radiation doses based on dosimetry calculations.	[86].
[^68^Ga]Ga-PSMA-I&T PET/CT	Prostate cancer staging, restaging, and response assessment demonstrate promising results, particularly in cases of metastatic castration-resistant prostate cancer. Providing critical data to support clinical decision-making.	[87].
[^68^Ga]Ga-PSMA-11 PET/CT	The importance of ^68^Ga-PSMA-11 in the diagnostic evaluation of prostate cancer, particularly in the context of biochemical recurrence.	[88].
[^89^Zr]Zr-PSMA-617	Patients with metastatic castration-resistant prostate cancer may benefit significantly from this approach in terms of improved therapeutic results, treatment planning, and diagnosis accuracy. The advantages of therapeutic utility and imaging quality are probably more remarkable than the risks associated with higher radiation exposure.	[89].
[^18^F]F-PSMA-1007	Demonstrated high specificity and effective biodistribution in preclinical models for prostate cancer detection.	[90,91,92].
[^99m^Tc]Tc-PSMA-I&S	The compound was initially developed for radio-guided surgery and demonstrated slow whole-body clearance, leading to increasing lesion-to-background ratios over time. This characteristic makes it suitable for the intraoperative detection of PSMA-positive lymph node metastases. The study aimed to assess its diagnostic use in a large cohort of prostate cancer patients.	[64,93,94].

**Table 3:** Therapeutic applications.

Therapeutic Application	Description	Radiopharmaceuticals Used	Study Findings	References
PSMA Therapy	Targeted radioligand therapy using lutetium-177-labeled PSMA inhibitors for metastatic castration-resistant prostate cancer (mCRPC).	[^177^Lu]Lu-PSMA-617, [^177^Lu]Lu-PSMA-I&T	Clinical trials show significant PSA reduction and improve overall survival in mCRPC patients.	[49,99,100,101,102].
Alpha Emitter Therapy	Use of alpha-emitting radiopharmaceuticals for targeted therapy in advanced prostate cancer.	[^223^Ra]RaCl^2^, [211At]At-PSMA	Studies indicate improved survival rates and reduced bone pain in patients with bone metastases.	[44,68,103,104].
Combination Therapy	Combining radiopharmaceuticals with other treatment modalities (e.g., chemotherapy, immunotherapy) for enhanced efficacy.	[^177^Lu]Lu-PSMA with docetaxel	Preliminary results suggest synergistic effects, leading to better treatment responses and patient outcomes.	[87].
Theranostic Approach	Integration of diagnostic imaging and therapeutic applications using PSMA-targeted agents for personalized treatment.	[^68^Ga]Ga-PSMA for imaging, [^177^Lu]Lu-617 PSMA for therapy	Patients with high PSMA uptake on imaging showed better responses to subsequent therapy, guiding treatment decisions.	[105,106].
Radiolabeled Small Molecule	Use of radiolabeled small-molecule-targeting prostate cancer antigens for therapy.	^124^I/^131^I-labeled small molecule (MIP-1095)	Studies demonstrate targeted delivery of radiation to tumor sites, resulting in tumor shrinkage and improved survival.	[107].

**Table 4:** Clinical studies.

Clinical Studies	Findings	Reference
Therapeutic Efficacy	Promising outcomes in safety and efficacy in patients with advanced prostate cancer	[24,38].
Biodistribution Studies	Specific accumulation in prostate tumor lesions, indicating potential for targeted therapy	[38,99].
Versatile Approach	Various uses including imaging, therapy, and radio-guided surgery, contributing to widespread success	[38].

**Table 5:** Linkages used in radiolabeled PSMA ligand synthesis.

Linkage Type	Examples	Function	Application	Reference
Chelators	DOTA, NOTA, HBED	Form stable complexes with radiometals for efficient radiolabeling of PSMA ligands	Developing radiolabeled PSMA ligands for radionuclide therapy and PET imaging	[43,117].
Peptide Linkers	Gly-Lys, Gly-Tyr	Gly-Tyr designed to be cleaved by brush border enzymes in proximal tubule cells, facilitating rapid excretion of radiometabolites	A promising strategy to reduce renal retention of LMW PSMA radioligands	[118].
Spacer Molecules	PEG spacers, alkyl linkers	Modulate distance between PSMA-binding domain and radionuclide, influencing binding affinity	Fine-tune pharmacokinetics and tumor-targeting properties of radiolabeled PSMA ligands	[77,119].
Bifunctional Chelating Agents (BFCAs)	DOTA-NHS ester, NOTA-NHS ester	Allow conjugation of PSMA-targeting molecules to radiometals through stable chelation	Facilitate coupling of PSMA ligands with radionuclides for molecular imaging and targeted therapy	[120,121].
Chemistry Linkers	DBCO, azide linkers	Enable rapid and specific conjugation of PSMA-targeting agents with radionuclides	Utilized for synthesis of radiolabeled PSMA ligands with improved radiolabeling kinetics and stability	[55,122].

**Table 6:** Potential side effects of low-molecular-weight PSMA ligand labeled with a radioisotope.

Side Effect	Description	Reference
Renal Toxicity	Concern due to significant radiation doses to the kidneys. Strategies like hydration and renal protectants may mitigate risk.	[47,70].
Xerostomia	Common dry mouth side effect, particularly with PSMA-targeted treatments. Potential toxicity in salivary and lacrimal glands.	[12,47].
Hematologic Toxicity	Reported thrombocytopenia and neutropenia. Fractionated regimens suggested to minimize hematologic toxicity.	[44,124].
Bone Marrow Toxicity	Patients with bone marrow metastases may experience grade 3 anemia, highlighting the importance of monitoring bone marrow activity.	[125].
Peripheral Neuropathy	Common side effect in patients undergoing PSMA-targeted radioligand treatment.	[9].
Other Potential Effects	Fatigue, nausea, and other potential side effects. Close monitoring is essential for prompt management.	[9].
Long-term Efficacy and Safety	Ongoing evaluation of anti-tumor activity and safety. Importance of individualized treatment plans and multidisciplinary care.	[9].

**Table 7:** Specific challenges in enhancing the affinity for PSMA in ligand design.

Aspect	Challenge	Reference
Specificity	Off-Target Binding: Achieving high affinity for PSMA while minimizing off-target binding to non-PSMA tissues is crucial to enhance the specificity of the ligand and reduce potential side effects. PSMA Heterogeneity: PSMA expression levels and isoform variations in different prostate cancer lesions can complicate ligand design, requiring effective strategies to target diverse PSMA profiles. Designing ligands that selectively target PSMA in cancer cells without binding to similar proteins or receptors in normal tissues is crucial to minimize off-target effects and toxicity.	[42,97].
Internalization	Endocytosis Efficiency: Designing ligands that bind strongly to PSMA on the cell surface and efficiently internalize into the target cells is essential for effectively delivering therapeutic payloads. Intracellular Trafficking: Understanding the intracellular fate of PSMA ligands post-internalization is critical to optimize drug release and maximize the therapeutic impact within cancer cells.	[44,77].
Pharmacokinetics	The pharmacokinetic properties of ligands, including absorption, distribution, metabolism, and excretion (ADME), significantly influence their effectiveness. Ligands must be designed to achieve optimal biodistribution to the tumor site while minimizing accumulation in non-target organs. Modifying specific amino acids in the peptide sequence can enhance stability and reduce susceptibility to enzymatic degradation, thereby prolonging the half-life of the peptides in circulation. Optimal Clearance: A critical challenge in ligand design is balancing the ligand’s affinity with its pharmacokinetic properties to ensure appropriate tissue distribution, clearance rates, and circulation times. Renal Excretion: Addressing the rapid renal clearance of small molecules to minimize background signals in the kidneys and bladder while maximizing tumor uptake is critical in ligand optimization.	[128,129,130].
Size and Structure	Molecular Weight: Finding the right balance between the molecular weight of the ligand and its affinity for PSMA is essential to optimize tumor penetration, biodistribution, and therapeutic efficacy. Structural Modifications: Adding linkers, spacers, or targeting moieties to a ligand to enhance its binding affinity and selectivity for PSMA could prove to be a challenging task due to the intricate interactions involved. PSMA is a type II transmembrane protein with a complex structure that includes an extracellular domain, a transmembrane region, and a short cytoplasmic tail. Designing ligands that can effectively bind to the extracellular domain while maintaining specificity and affinity is challenging due to the intricate folding and conformational dynamics of the protein.	[42,77,108].
Resistance Mechanisms	Emergence of Resistance: Anticipating and overcoming potential resistance mechanisms to PSMA-targeted therapies, such as alterations in PSMA expression or downstream signaling pathways, is crucial for long-term treatment success. Combination Strategies: Developing combination therapies that target complementary pathways or mechanisms alongside PSMA to overcome resistance and enhance treatment outcomes presents a multifaceted challenge in ligand design. Addressing these challenges in ligand design through innovative strategies, structural optimization, and a deep understanding of PSMA biology is essential to enhance the affinity, specificity, and therapeutic potential of PSMA-targeted therapies for prostate cancer and other PSMA-expressing malignancies.	[44,77,131].
Delivery Mechanisms	Effective delivery systems are required to ensure that peptides reach their target sites in sufficient concentrations. The development of reliable delivery systems, such as nanoparticles, is essential to overcome barriers related to peptide stability and targeting.	[132,133].

## Data Availability

There are no data outside those reported in this article.

## Glossary

- Biodistribution: The distribution of a substance, such as a drug or radiopharmaceutical, within the body after administration.
- Clinical Trials: Research studies that test new treatments or interventions in people to determine their safety and effectiveness.
- Diagnostic Imaging: Techniques used to visualize the body’s interior for clinical analysis, including methods like MRI, CT scans, and PET scans.
- Half-Life: The time required for half of a radionuclide to decay is crucial for determining the duration of radiopharmaceutical activity in the body.
- Incidence Rate: The number of new disease cases in a specific population during a defined period, often expressed per 100,000 individuals.
- Immunotherapy: A type of cancer treatment that helps the immune system fight cancer.
- Linear Energy Transfer (LET): The average amount of energy deposited by radiation per unit of distance traveled by an ionizing particle. It is measured in kilo electron volts per micrometer (keV/μm).
- Low-Molecular-Weight PSMA Ligands: Small molecules that specifically bind to PSMA and are used in imaging and therapy for prostate cancer.
- Metastatic Castration-Resistant Prostate Cancer (mCRPC): A stage of prostate cancer that has spread beyond the prostate and is no longer responsive to hormonal therapy aimed at lowering testosterone levels.
- Overall Survival (OS): A measure of the length of time from diagnosis or the start of treatment that patients are still alive.
- Personalized Medicine: An approach to patient care that tailors treatment based on individual characteristics, including genetics and specific disease characteristics.
- Pharmacokinetics: The study of how radiopharmaceuticals are absorbed, distributed, metabolized, and excreted in the body.
- Prevalence: The total number of cases of a disease in a population at a given time, regardless of when the disease first appeared.
- Prognosis: The likely course and outcome of a disease, including chances of recovery and survival.
- Prostate Cancer: A type of cancer that occurs in the prostate, a small walnut-shaped gland that produces seminal fluid in men.
- Quality of Life: A measure of an individual’s overall well-being, including physical, mental, and social health, particularly in the context of chronic illness.
- Radioligand Therapy: A treatment that uses radiolabeled ligands to target and destroy cancer cells, often used in conjunction with imaging techniques.
- Radionuclide: A radioactive atom emitting radiation; used in radiopharmaceuticals for diagnosis or therapy.
- Supportive Care: Services and treatments that help manage symptoms and improve the quality of life for patients with serious illnesses.
- Therapeutic Agents: Medications or treatments used to manage or cure diseases.
- Theranostics: A field of medicine that combines therapeutic and diagnostic capabilities, often using targeted therapies monitored through imaging.
